# Dysfunction of Mitochondrial Ca^2+^ Regulatory Machineries in Brain Aging and Neurodegenerative Diseases

**DOI:** 10.3389/fcell.2020.599792

**Published:** 2020-12-18

**Authors:** Hyunsu Jung, Su Yeon Kim, Fatma Sema Canbakis Cecen, Yongcheol Cho, Seok-Kyu Kwon

**Affiliations:** ^1^Center for Functional Connectomics, Brain Science Institute, Korea Institute of Science and Technology, Seoul, South Korea; ^2^Division of Life Sciences, Korea University, Seoul, South Korea; ^3^Department of Biomedical Sciences, College of Medicine, Korea University, Seoul, South Korea; ^4^Division of Bio-Medical Science & Technology, KIST School, Korea University of Science and Technology (UST), Seoul, South Korea

**Keywords:** mitochondria, calcium regulation, aging, neurodegenerative disease, synaptic regulation

## Abstract

Calcium ions (Ca^2+^) play critical roles in neuronal processes, such as signaling pathway activation, transcriptional regulation, and synaptic transmission initiation. Therefore, the regulation of Ca^2+^ homeostasis is one of the most important processes underlying the basic cellular viability and function of the neuron. Multiple components, including intracellular organelles and plasma membrane Ca^2+^-ATPase, are involved in neuronal Ca^2+^ control, and recent studies have focused on investigating the roles of mitochondria in synaptic function. Numerous mitochondrial Ca^2+^ regulatory proteins have been identified in the past decade, with studies demonstrating the tissue- or cell-type-specific function of each component. The mitochondrial calcium uniporter and its binding subunits are major inner mitochondrial membrane proteins contributing to mitochondrial Ca^2+^ uptake, whereas the mitochondrial Na^+^/Ca^2+^ exchanger (NCLX) and mitochondrial permeability transition pore (mPTP) are well-studied proteins involved in Ca^2+^ extrusion. The level of cytosolic Ca^2+^ and the resulting characteristics of synaptic vesicle release properties are controlled *via* mitochondrial Ca^2+^ uptake and release at presynaptic sites, while in dendrites, mitochondrial Ca^2+^ regulation affects synaptic plasticity. During brain aging and the progress of neurodegenerative disease, mitochondrial Ca^2+^ mishandling has been observed using various techniques, including live imaging of Ca^2+^ dynamics. Furthermore, Ca^2+^ dysregulation not only disrupts synaptic transmission but also causes neuronal cell death. Therefore, understanding the detailed pathophysiological mechanisms affecting the recently discovered mitochondrial Ca^2+^ regulatory machineries will help to identify novel therapeutic targets. Here, we discuss current research into mitochondrial Ca^2+^ regulatory machineries and how mitochondrial Ca^2+^ dysregulation contributes to brain aging and neurodegenerative disease.

## Introduction

Mitochondria affect cellular functions *via* their roles in ATP production, lipid synthesis, reactive oxygen species (ROS) generation, and Ca^2+^ regulation. Recent studies of mitochondria-dependent Ca^2+^ handling have revealed the molecular identities of Ca^2+^-control components, including the mitochondrial calcium uniporter (MCU) and its auxiliary subunits (Mammucari et al., 2017). Furthermore, the development of enhanced Ca^2+^ sensors has enabled subcellular investigations of how mitochondria contribute to synaptic transmission. In addition, mitochondrial matrix-targeting sequence-tagged genetically encoded calcium indicators (GECIs) have allowed direct monitoring of mitochondrial Ca^2+^ dynamics (Kwon et al., 2016a).

In aged animals and humans, mitochondrial functional impairment is a key hallmark of brain aging (Grimm and Eckert, 2017; Mattson and Arumugam, 2018; Muller et al., 2018). Alzheimer’s disease (AD), Parkinson’s disease (PD), Huntington’s disease (HD), and other aging-related neurodegenerative diseases also show mitochondrial defects. However, the detailed molecular mechanisms underlying these defects, particularly those related to mitochondrial Ca^2+^, have not yet been studied in depth.

Here, we describe mitochondrial Ca^2+^-related features and unveiled mitochondrial Ca^2+^ regulatory molecular mechanisms of brain aging and neurodegenerative disease models, and discuss experimental methods and controversies within the current research.

## Mitochondrial Ca^2+^ Regulatory Components and Their Physiological Roles in Neurons

Ca^2+^ ions enter neurons through ionotropic glutamate receptors and voltage-dependent Ca^2+^ channels, with the imported Ca^2+^ then affecting various cellular processes, including the modulation of synaptic strength and Ca^2+^-mediated cell death (Ghosh and Greenberg, 1995). At the presynapse, Ca^2+^ triggers synaptic vesicle exocytosis, and residual Ca^2+^ alters synaptic release properties toward asynchronous release. Moreover, short-term synaptic plasticity can be controlled by presynaptic Ca^2+^ dynamics, while in dendrites, Ca^2+^ influences various signaling cascades involved in long-term synaptic plasticity and gene transcription (Hayashi and Majewska, 2005; Higley and Sabatini, 2008; Sudhof, 2012; Kaeser and Regehr, 2014; Kwon et al., 2016a).

Cytosolic Ca^2+^ is controlled by plasma membrane Ca^2+^ pumps and intracellular organelles, including mitochondria and the endoplasmic reticulum (ER). The ER imports Ca^2+^ through sarco/endoplasmic Ca^2+^ ATPase and releases it *via* ryanodine receptors or IP_3_ receptors (IP_3_Rs) (Verkhratsky, 2005). ER is partially tethered to mitochondria by mitochondria-associated membrane (MAM) or mitochondria-ER contact sites (MERCs) proteins such as Mitofusin 2, Sigma-1 receptor, vesicle-associated membrane protein-associated protein B (VAPB)/protein tyrosine phosphatase-interacting protein 51 (PTPIP51), IP3R/glucose-regulated protein (Grp75)/Voltage-dependent anion-selective channel 1 (VDAC1), and PDZ domain containing 8 (PDZD8), enabling ER-to-mitochondria Ca^2+^ transfer (Rapizzi et al., 2002; Szabadkai et al., 2006; Hayashi and Su, 2007; De Vos et al., 2012; Hirabayashi et al., 2017). The involvement of MAM in neurodegenerative diseases is a major topic in the field, but previous reviews wonderfully covered this scope (Paillusson et al., 2016; Liu and Zhu, 2017; Area-Gomez et al., 2018; Bernard-Marissal et al., 2018; Gomez-Suaga et al., 2018; Lau et al., 2018). Therefore, only a part of studies using direct observation of mitochondrial Ca^2+^ dynamics will be discussed here.

Several Ca^2+^ regulatory proteins have been identified in the outer and inner mitochondrial membranes. VDACs located in the outer mitochondrial membrane (OMM) are responsible for importing various ions and metabolites (Colombini, 2016). In the inner mitochondrial membrane, MCU mediates mitochondrial membrane potential-dependent Ca^2+^ influx into the mitochondrial matrix (Kirichok et al., 2004; Baughman et al., 2011; De Stefani et al., 2011). Reduced MCU-dependent Ca^2+^ uptake at presynaptic sites elevates cytosolic Ca^2+^ and alters short-term synaptic plasticity and synchronous release (Kang et al., 2008; Kwon et al., 2016b). In addition, a recent study showed upregulation of mitochondrial fission and dendritic mitochondrial Ca^2+^ transients following chemically induced long-term potentiation (LTP), with the interference of fission impairing mitochondrial Ca^2+^ uptake and LTP (Divakaruni et al., 2018).

Mitochondrial calcium uniporter forms complexes with other proteins, which regulate its opening dynamics (Mammucari et al., 2017; Pallafacchina et al., 2018). Mitochondrial calcium uptake protein 1/2/3 (MICU1/2/3) are the first MCU binding proteins to be characterized. MICU1 and MICU2 serve as molecular gatekeepers that negatively regulate MCU under low Ca^2+^ but positively regulate it under high cytosolic Ca^2+^ (Csordas et al., 2013; Patron et al., 2014; Liu et al., 2016). MICU3 is abundant in the brain and enhances mitochondrial Ca^2+^ uptake, with silencing of MICU3 in cortical neurons causing a reduction in stimulation-induced mitochondrial Ca^2+^ levels (Patron et al., 2019). Furthermore, the presynaptic MICU3-dependent increase in Ca^2+^ sensitivity allows MCU to open without Ca^2+^ release from ER and facilitates Ca^2+^-mediated mitochondrial ATP production and synaptic vesicle endocytosis (Ashrafi et al., 2020).

Essential MCU regulator (EMRE) is another MCU complex protein that bridges MCU and MICU1 and regulates the level of Ca^2+^ in the mitochondrial matrix. In addition, recent unveiled structural features of EMRE show that it triggers dimerization of MCU-EMRE complex and controls pore opening (Sancak et al., 2013; Vais et al., 2016; Wang et al., 2019). The MCU paralog MCUb exerts an inhibitory effect on MCU, with overexpression of MCUb completely abolishing MCU currents (Raffaello et al., 2013; Mammucari et al., 2017). Mitochondrial calcium uniporter regulator 1 (MCUR1) is a scaffold factor, whose absence results in the failure of MCU to form a complex (Tomar et al., 2016; Supplementary Figure 1).

The mitochondrial Na^+^/Ca^2+^ exchanger (NCLX) is one of the primary Ca^2+^ efflux units in mitochondria (Palty et al., 2010). Genetic ablation of NCLX increases Ca^2+^ retention in mitochondria and causes mitochondria-dependent cell death (Luongo et al., 2017). Also, H^+^/Ca^2+^ exchanger is considered as a Ca^2+^ efflux component, although its molecular identity is arguable (Jiang et al., 2009; De Marchi et al., 2014). Additional Ca^2+^ release-associated protein, mitochondrial permeability transition pore (mPTP), is activated by Ca^2+^ overload and ROS, leading to apoptosis or necrosis (Giorgi et al., 2018). This pore has been mainly studied under pathological conditions, including aging and neurodegenerative diseases, and the roles of core components including ATP synthase, cyclophilin D (CyPD), and the adenine nucleotide translocators (ANTs), are recently updated, although there are debates (Kokoszka et al., 2004; Bonora et al., 2013; Alavian et al., 2014; Karch and Molkentin, 2014; Raffaello et al., 2016; He et al., 2017; Rottenberg and Hoek, 2017; Zhou et al., 2017; Bernardi, 2018; Muller et al., 2018; Carroll et al., 2019; Karch et al., 2019).

## Mitochondrial Ca^2+^ Dyshomeostasis in Aged Brains

Dysregulation of Ca^2+^ homeostasis is one of the hallmarks of brain aging (Mattson and Arumugam, 2018), with impaired Ca^2+^ control in aged brains resulting in various cellular and physiological deficits. Hippocampal CA1 pyramidal neurons in aged animals show elevated Ca^2+^ currents, as confirmed by Ca^2+^ imaging using multiple Ca^2+^ fluorophores (Landfield and Pitler, 1984; Disterhoft et al., 1996; Verkhratsky et al., 1998; Thibault et al., 2001; Lessmann et al., 2003). Age-associated Ca^2+^ changes have also been observed in other brain regions and in peripheral nerves (Verkhratsky et al., 1998).

Age-dependent dysregulation of Ca^2+^ results from various molecular changes, including increased voltage-gated Ca^2+^ channel expression, reduced Ca^2+^ binding protein expression, and impaired mitochondrial and ER Ca^2+^ handling (Mattson and Arumugam, 2018). Ca^2+^ isotope uptake by isolated synaptosomal mitochondria is significantly reduced in aged rat brains (Leslie et al., 1985). In addition, cytosolic Ca^2+^ dynamics in aged rodent brain slices or acutely dissociated neurons have been monitored using chemical Ca^2+^ dyes, such as Fura-2. Use of this dye in combination with a mitochondrial membrane potential indicator or mitochondrial uncoupler has revealed that the potential is disrupted in aged neurons, resulting in a decrease in mitochondrial Ca^2+^ uptake and an elevation of cytosolic Ca^2+^ upon stimulation (Xiong et al., 2002; Murchison et al., 2004). Mitochondrial Ca^2+^ buffering is also reduced in aged Rhesus monkeys, shown using isolated putamen mitochondria (Pandya et al., 2015).

## Altered Mitochondrial Ca^2+^ Dynamics in AD

Ca^2+^ dysregulation is a common feature of several neurodegenerative diseases, including AD and PD (Beal, 1998; Zundorf and Reiser, 2011; Liao et al., 2017; Pchitskaya et al., 2018). Disruption of Ca^2+^ homeostasis and mitochondrial Ca^2+^ overload have been observed before pathological features of these diseases appear, highlighting the importance of neuronal Ca^2+^ regulation (Lesne et al., 2008; Surmeier et al., 2017).

AD is characterized by the accumulation of amyloid beta (Aβ) peptide, which is produced by abnormal proteolytic cleavage of amyloid precursor protein (APP), as well as by the formation of neurofibrillary tangles composed of tau protein and by neuronal loss, leading to learning and memory impairment (Oddo et al., 2003; Mattson et al., 2008). Mutations in APP or in the γ-secretase components presenilin1 and 2 (PSEN1/2) are the most well-characterized alterations contributing to dominantly inherited familial AD (FAD) (Chen et al., 2017; Muller et al., 2018).

Increased Aβ expression in FAD models or exogenous application of Aβ leads to elevated cytosolic Ca^2+^. In the past two decades, multiple underlying mechanisms have been suggested, including mitochondrial Ca^2+^ dysregulation (Du et al., 2008; Supnet and Bezprozvanny, 2010; Jadiya et al., 2019; Calvo-Rodriguez et al., 2020). *In-vivo* Ca^2+^ imaging with mitochondria-targeted Förster resonance energy transfer (FRET)-based GECI has directly demonstrated an Aβ-dependent mitochondrial Ca^2+^ increase in mouse cortex. This upregulation was observed prior to neuronal death, with blockade of MCU restoring the mitochondrial Ca^2+^ level in the APP/PS1 mutant mouse model (Calvo-Rodriguez et al., 2020).

Brain levels of NCLX protein are significantly reduced in human AD patients and in 3xTg-AD triple mutant mice (expressing mutations in APP, presenilin 1, and tau). Mutant APP-expressing-N2a cells also show decreased NCLX expression, resulting in impaired mitochondrial Ca^2+^ extrusion, consistent with the increased mitochondrial Ca^2+^ transients revealed by the mitochondria-localized GECI mito-R-GECO1. Alleviation of mitochondrial Ca^2+^ overload by NCLX expression in 3xTg-AD mice rescues cognitive decline and AD-related pathology (Jadiya et al., 2019). Another Ca^2+^ extrusion-related molecule, CypD, which is part of mPTP, is known to interact with mitochondrially transported Aβ. Inhibition or genetic ablation of CypD protects neurons from Aβ-triggered cell death and rescues impaired LTP and deficits in spatial learning and memory (Du et al., 2008).

**FIGURE 1:**
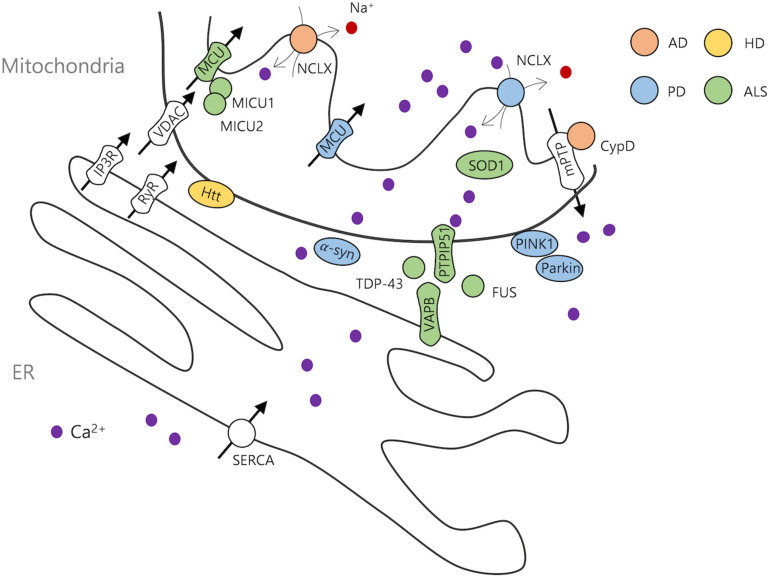
Neurodegenerative disease-related ER and mitochondrial proteins. AD, Alzheimer’s disease; PD, Parkinson’s disease; HD, Huntington’s disease; ALS, amyotrophic lateral sclerosis.

Mitochondrial Ca^2+^ overload in AD can also result from impaired ER–mitochondria communication. Previous studies suggest that ER–mitochondria contacts are increased in AD models, promoting Ca^2+^ transfer to mitochondria (Zampese et al., 2011; Area-Gomez et al., 2012; Hedskog et al., 2013; Calvo-Rodriguez et al., 2019). ER-to-mitochondria Ca^2+^ transfer has been monitored using the mitochondria-localized chemical dye Rhod-5N or a mitochondrial matrix- or OMM-targeted protein Ca^2+^ sensors (Zampese et al., 2011; Hedskog et al., 2013; Calvo-Rodriguez et al., 2019). Opposite to these, some studies using electron microscopy (EM) and fluorescent imaging have reported reduced ER-mitochondria contacts in AD animal models and patients (Sepulveda-Falla et al., 2014; Martino Adami et al., 2019; Lau et al., 2020; Figure 1).

Apolipoprotein E4 (ApoE4) is the major risk factor for sporadic AD and it can increase ER–mitochondria contacts (Tambini et al., 2016; Orr et al., 2019). ApoE4-expressing cells show higher cytosolic and mitochondrial Ca^2+^, and given that ApoE4 expression alters neuronal MAM-tethering protein composition, this could explain enhanced MAM activity in sporadic AD (Orr et al., 2019; Table 1).

**TABLE 1:** Ca^2+^ dynamics in neurodegenerative disease models.

Disease	Model		Ca^2+^ level		Ca^2+^ indicator		Ca^2+^ inducer	ER-mito contact	References
			Cyto	Mito	Cyto	Mito			
AD	Aβ	APP/PS1 mut *in-vivo* cortex	↑	↑		mtYellow Cameleon3.6			Calvo-Rodriguez et al., 2020
	Aβ oligomer	Hippocampal neuron (DIV15-21)	↑	↓	Fura-2	Rhod-5N	Caffeine, ACh	↑	Calvo-Rodriguez et al., 2019
	APP_*swe*_ 3xTG-AD	Neuroblastoma N2a cell	↑	↑	Fura4-AM	mito-R-GECO1	KCl		Jadiya et al., 2019
	Aβ	APP_*swe/Lon*_ SH-SY5Y	↔	↑	Cyt-AEQ	mit-AEQ	Bradykinin	↑	Hedskog et al., 2013
	PS2 (WT, T122R)	SH-SY5Y		↑ (ER-mito transfer)	Cyt-AEQ, N33D1cpv	4mtD1cpv, mit-AEQ	Bradykinin	↑	Zampese et al., 2011
	APOE4	Neuroblastoma N2a cell	basal ↔	↑	Fura-2 AM	Rhod-2AM	CaCl_2_, Thapsigargin	↑ (MAM protein level)	Orr et al., 2019
PD	α–synuclein (WT)	SH-SY5Y HeLa cell	↔	↑	Cyt-AEQ	mit-AEQ	Bradykinin, Histamine	↑	Cali et al., 2012
	α–synuclein (WT, A53T, A30P)	SH-SY5Y Neuron derived from patient iPSC		↓		Rhod2-AM	Oxo-M	↓	Paillusson et al., 2017
	PINK1 mutant	Drosophila	↔	↑	GCaMP	mito-GCaMP, Rhod2-AM		↑	Lee et al., 2018
	PINK1 KD / KO	SH-SY5Y human neuron mouse neuron	↑	↑ (Capacity ↓)	Fluo-4, Fura-2		KCl, Ca-NPEGTA		Gandhi et al., 2009
	PINK1 KO	Isolated mitochondria		Capacity ↓		Extra mitochondria Ca^2+^: Calcium Green 5N	KCl		Akundi et al., 2011
	Parkin KD / mutant	Drosophila Patient fibroblast	↔	↓	Cyt-AEQ	mit-AEQ	Histamine ATP	↓	Basso et al., 2018
	Parkin KO / mutant	PARK2 KO mouse Patient fibroblast	↓	↑	Fura-2	N33-D1cpv, pericam-mt	Bradykinin, ATP, Histamine	↑	Gautier et al., 2016
	LRRK2 (G2019S, R1441C)	Cortical neuron patient fibroblast	↑	↑	RCaMP	mt-GCaMP6m	KCl		Verma et al., 2017
HD	HTT (YAC72)	Isolated mitochondria (YAC72 brain)	↑ (slower recovery)	Capacity ↓		Extra-mitochondrial Ca^2+^ : Calcium Green 5N	CaCl_2_		Panov et al., 2002
	HTT (YAC128)	Isolated mitochondria, striatal neuron	↑	Capacity ↑	Fura-2FF-AM	Extra-mitochondrial Ca^2+^ using electrode	CaCl_2_ (w/o BSA), glutamate		Pellman et al., 2015
	HTT (YAC128)	Isolated forebrain mitochondria		↑		Extra-mitochondrial Ca^2+^ : Calcium Green 5N	CaCl_2_		Oliveira et al., 2007
	R6/2 mice			↑					
	Hdh150 knock-in mice			↔					
	STHdhQ111	Striatal cell line	↔↑(Bradykinin)	↓↔ (low Ca^2+^)	Fura-2AM	mit-AEQ	ATP, Bradykinin, Ca^2+^		Lim et al., 2008
	STHdhQ111	Striatal cell line	↔	↓	Fluo-3AM	Rhod-2AM	Thapsigargin		Quintanilla et al., 2013
	HTT (YAC128)	Medium spiny neuron (MSN)	↑		Fura-2		Glutamate		Tang et al., 2005
	HTT (YAC128), HD patient	MEF, MSN, patient fibroblast		↑		2mt-cameleon	Bradykinin, DHPG		Wang et al., 2013
ALS	SOD1 (G37R)	Neuroblastoma N2a cell	↑	↓	Cyt-AEQ	mit-AEQ	Bradykinin		Coussee et al., 2011
	SOD1 (G93A)	Motor neuron	↓ (release from mito)		Fura-2AM		FCCP		Tadic et al., 2019
	SOD1 (G93A)	Motor neuron	↑	↓	Fura-2AM	Rhod-2AM	Glutamate		Kruman et al., 1999
	SOD1 (G93A)	Motor neuron	↑	↑	Fura-2AM	mt-pericam	No inducer		Tradewell et al., 2011
	SOD1 (G93A)	Isolated spinal cord mitochondria		↓endstage & presymptomatic		Calcium Green 5N			Parone et al., 2013
	SOD1 (G37R, G85R)			↓endstage ↔presymptomatic					
	TDP43 (M337V, Q331K, A382T, G348C)	HEK293	↑	↓	Fluo-4AM	Rhod-2AM	Oxo-M	↓	Stoica et al., 2014
	FUS (R521C, R518K)	HEK293	↑	↓	Fluo-4AM	Rhod-2AM	Oxo-M	↓	Stoica et al., 2016
	C9ORF72, TARDBP (M337V, I383T)	Patient fibroblast derived MN	↑ (recovery time)	↓	Fura-2AM	Rhod-2AM	KCl, Glutamate		Dafinca et al., 2020

*Disease models show differential Ca^2+^ dynamics depending on disease models, Ca^2+^ sensors, and stimulation condition. Disease models: 3xTG-AD, Presenilin 1 (Psen1, M146V homozygous knock-in), amyloid beta precursor protein (APPswe, K670N/M671L transgene) and microtubule associated protein tau (MAPT, P301L transgene); APP_*swe/Lon*_, Swedish (K670N/M671L) and London (V717I) mutations; YAC models, expanded number of polyQ in Htt; R6/2 mice, expressing a short N-terminal fragment of human Htt with 150 polyQ; Hdh150 knock-in mice, full-length Htt with 150 polyQ; STHdhQ111, striatal cell lines from HD knock-in mouse model; Ca^2+^ sensors: AEQ, aequorin (chemiluminescence-based genetically encoded Ca^2+^ sensor); D1cpv, FRET-based Ca^2+^ sensor (N33 for outer mitochondrial membrane, 4 mt for mitochondrial matrix); Etc: Ach, Acetylcholine; DHPG, (*RS*)-3,5-dihydroxyphenylglycine, a potent agonist of group I metabotropic glutamate receptors; MEF, mouse embryonic fibroblast.*

Furthermore, mitochondria contribute to the presynaptic defects observed in AD. Increased insulin-like growth factor-1 receptor (IGF-1R) levels are found in AD patient and mouse model brain samples (Moloney et al., 2010; Zhang et al., 2013). IGF-1R regulates synaptic transmission by modulating presynaptic mitochondrial Ca^2+^ buffering and ATP production, as measured using the mitochondria-targeted GECI and the ATP sensor, although detailed mechanisms are not known. Interestingly, inhibition of IGF-1R reverses altered synaptic release in an APP/PS1 mutant model (Gazit et al., 2016).

## PD-Related Mitochondrial Ca^2+^ Dysfunction

Parkinson’s disease is characterized at the cellular level by dopaminergic neuron loss in the substantia nigra. Several genes contributing to PD pathogenesis have been identified, including α-synuclein, leucine-rich repeat kinase 2 (LRRK2), PTEN-induced kinase 1 (PINK1), and parkin (Abou-Sleiman et al., 2006; Ferreira and Massano, 2017). Ca^2+^ regulation is especially important for dopaminergic neurons because of their steady and autonomous pacemaker function (Chan et al., 2007; Guzman et al., 2010).

Mutation of α-synuclein and its aggregation into Lewy bodies are well-known pathological processes in PD. Interestingly, α-synuclein is localized to ER, mitochondria, and MAM and contributes to regulating ER–mitochondria communication (Li et al., 2007; Cali et al., 2012; Guardia-Laguarta et al., 2014). In one study, overexpression of WT or mutant α-synuclein in HeLa and SH-SY5Y cells was found to increase mitochondrial Ca^2+^ by enhancing ER–mitochondria interaction (Cali et al., 2012, 2019). However, another study using mutant α-synuclein-overexpressing cells produced conflicting results in terms of ER–mitochondria interactions (Guardia-Laguarta et al., 2014). Furthermore, overexpression of α-synuclein (WT/mutant) in SH-SY5Y cells disturbed the interaction between VAPB and PTPIP51, and this was accompanied by reduced ER-to-mitochondria Ca^2+^ transfer (Paillusson et al., 2017). High dose of WT/mutant α-synuclein can form the aggregates, and this in turn reduces ER-mitochondria contacts (Cali et al., 2012, 2019). Therefore, the dose-dependent effect could be the possible cause of discrepancies (Figure 1).

PINK1, a mitochondrial serine/threonine kinase, and parkin, an E3 ubiquitin ligase, are proposed to underlie mitochondrial quality control, with mutations in either gene highly related to PD (Narendra and Youle, 2011; Pickrell and Youle, 2015). Dopaminergic neuron-specific mitochondrial Ca^2+^ imaging with mito-GCaMP, a mitochondria-targeted GECI, in *Drosophila* PD models revealed elevated mitochondrial Ca^2+^. Pharmacological and genetic inhibition of IP_3_R and MCU restore mitochondrial Ca^2+^ and dopaminergic neuron loss (Lee et al., 2018). In contrast, due to negative regulation of NCLX, PINK1-deficient cortical neurons show reduced mitochondrial Ca^2+^ capacity and higher mitochondrial Ca^2+^ accumulation (Gandhi et al., 2009). Similarly, purified mitochondria from PINK1^–/–^ mouse brain show a significantly decreased mitochondrial Ca^2+^ buffering capacity (Akundi et al., 2011).

Primary fibroblasts from PD patients with parkin mutations had reduced mitochondrial Ca^2+^ uptake due to loosened ER–mitochondria connectivity (Basso et al., 2018). Contrary to these results, other studies found that fibroblasts from Parkin-deficient mice or from PD patients with a parkin mutation show increased ER–mitochondria contacts (Gautier et al., 2016). In addition, OMM- and matrix-targeted Ca^2+^ sensors revealed higher ER-mitochondrial Ca^2+^ transfer (Gautier et al., 2016).

Dendrite shortening in LRRK2 mutant models is a well-known change related to mitochondrial dysfunction (Cherra et al., 2013). Abnormal mitochondrial function results from the stimulation-induced increase in mitochondrial Ca^2+^, which is accompanied by upregulated MCU expression. Interestingly, chemical inhibition or knockdown of MCU successfully restores neurite length (Verma et al., 2017; Table 1).

## Dysregulation of Mitochondrial Ca^2+^ in Other Neurodegenerative Diseases

HD is a hereditary neurodegenerative disease characterized by involuntary movements, psychiatric abnormalities, and dementia. The major pathogenic features of the disease are progressive striatal neuronal loss, particularly of GABAergic medium spiny neurons, and extension of the N-terminal polyglutamine (polyQ) stretch of the huntingtin protein (Walker, 2007; Brustovetsky, 2016).

In the mutant huntingtin transgenic mouse brain, polyQ-stretched huntingtin is associated with the mitochondrial membrane (Choo et al., 2004). Interestingly, deficits in mitochondrial Ca^2+^ buffering have been observed after applying Calcium Green-5N to isolated mitochondria from HD mouse brain to monitor extramitochondrial Ca^2+^ (Panov et al., 2002). Live imaging of HD model striatal cell lines with mitochondria-targeted aequorin or Rhod-2AM indicates that mitochondria are able to handle a low Ca^2+^ challenge, but that a higher Ca^2+^ concentration disrupts their buffering ability (Lim et al., 2008; Quintanilla et al., 2013).

However, other studies demonstrated increased Ca^2+^ uptake capacity in isolated mitochondria from HD model mouse forebrains (Oliveira et al., 2007; Pellman et al., 2015). Furthermore, mitochondrial Ca^2+^ influx is higher in primary medium spiny neurons of HD model mice (Tang et al., 2005) and in fibroblasts from HD patients (Wang et al., 2013), which leads to cell death or mitochondrial DNA damage. Interestingly, this excitotoxicity is prevented by MCU or mPTP inhibition (Tang et al., 2005; Figure 1 and Table 1).

Amyotrophic lateral sclerosis (ALS) is a neurodegenerative disease characterized by progressive muscle paralysis resulting from the degeneration of upper and lower motor neurons. ALS exhibits multiple pathogenic features, including oxidative stress, mitochondrial dysfunction, and protein dysfunction of 43-kDa transactivating response region binding protein (TDP-43) and cytoplasmic Cu^2+^/Zn^2+^-superoxide dismutase 1 (SOD1) (Ferraiuolo et al., 2011; Lee et al., 2011; Tadic et al., 2014).

Monitoring with mitochondria-/ER-targeted ratiometric sensor proteins and Fura-2 identified elevated levels of mitochondrial, ER, and cytosolic Ca^2+^ in the motor neurons of ALS mutant transgenic mice (SOD1^*G93A*^) (Tradewell et al., 2011). However, in another study, Rhod-2- and Fura-2-based Ca^2+^ imaging showed significantly decreased mitochondrial Ca^2+^ uptake and increased cytosolic Ca^2+^ in SOD1^*G93A*^ mice motor neurons (Kruman et al., 1999). Decreased mitochondrial Ca^2+^ buffering capacity in SOD1^*G93A*^-expressing mice can be restored by CypD deletion, which regulates mPTP opening, suppressing cell death (Parone et al., 2013). Other ALS mutant (SOD1^*G37R*^-overexpressing) N2a cells also show significantly reduced mitochondrial Ca^2+^ uptake and elevated cytosolic Ca^2+^ (Coussee et al., 2011).

Multiple studies suggest that specific molecular processes underlie ALS progression, but their findings are contentious. Hypoglossal motor neurons of SOD1^*G93A*^-transgenic mice show upregulated MCU and MICU1 expression at the end stage of the disease (P115–140) (Fuchs et al., 2013). However, in symptomatic cervical spinal cord motor neurons, MCU level is significantly decreased (Tadic et al., 2019).

Other ALS-associated genes have also been identified, including TDP-43, fused in sarcoma (FUS), VAPB, and expanded hexanucleotide repeats in intron 1 of the encoding chromosome 9 open reading frame 72 (C9ORF72). The OMM protein PTPIP51 is a known binding partner of the ER protein VAPB. VAPB–PTPIP51 interaction in mouse motor neurons is disrupted by overexpression of ALS mutant or wild-type TDP-43 and FUS, also leading to disruption of Ca^2+^ homeostasis in HEK293T cells (Stoica et al., 2014, 2016). ALS patient fibroblast-derived motor neurons with C9ORF72 and TDP-43 mutations show delayed clearance of cytosolic Ca^2+^, lower mitochondrial buffering capacity, and imbalance of MICU1 and MICU2 expression (Dafinca et al., 2020) (Figure 1 and Table 1).

## Discussion

In summary, brain aging and neurodegenerative diseases involve mitochondria- and ER-mitochondria contact-related Ca^2+^ regulatory defects. These alterations have been revealed using various experimental methods, including electrophysiological recording and live imaging. However, large part of *in-vitro* studies for neurodegenerative diseases have performed using cell lines and patient-derived fibroblasts rather than neurons. In addition, Ca^2+^ signals were triggered by various chemicals, and most conditions are not neurophysiological (Table 1). Depending on tissues and brain regions, mitochondrial Ca^2+^ uptake capacity and regulatory components can be different (Markus et al., 2016; Vecellio Reane et al., 2016; Patron et al., 2019). Therefore, application of recently advanced genetically encoded Ca^2+^ sensors for specific organelles will provide more precise neuron type-specific data (Kwon et al., 2016a).

Finally, mitochondrial Ca^2+^ regulatory molecular mechanisms have recently been revealed, with some studies showing that the composition of the MCU complex can change during disease progress and differ between mutant types (Table 1). Thus, revealing the detailed pathophysiological mechanisms of mitochondrial defects at the molecular level could lead to novel therapeutic targets that are specific for particular mutations and disease stages.

## Author Contributions

HJ, SYK, FC, and S-KK wrote the manuscript and created the figures and table. S-KK and YC provided guidance and edited the manuscript. All authors contributed to the article and approved the submitted version.

## Conflict of Interest

The authors declare that the research was conducted in the absence of any commercial or financial relationships that could be construed as a potential conflict of interest.

## Supplementary Material

Supplementary Figure 1 MCU complex machinery. When the cytosolic Ca^2+^ level is low, MICU1/MICU2 heterodimer keeps MCU as a closed-form, whereas in high Ca^2+^ concentration, their conformational change helps MCU allow Ca^2+^ influx toward mitochondrial matrix. Otherwise, MICU1/MICU3 heterodimer has less gatekeeping function than MICU1/MICU2 dimer, which leads to opening the MCU complex in lower Ca^2+^ condition.
