# Supplementary figures and images for: Dysfunction of Mitochondrial Ca2+ Regulatory Machineries in Brain Aging and Neurodegenerative Diseases
Source: Front Cell Dev Biol. 2020 Dec 18;8:599792. doi: 10.3389/fcell.2020.599792 (PMC7775422; doi:10.3389/fcell.2020.599792)

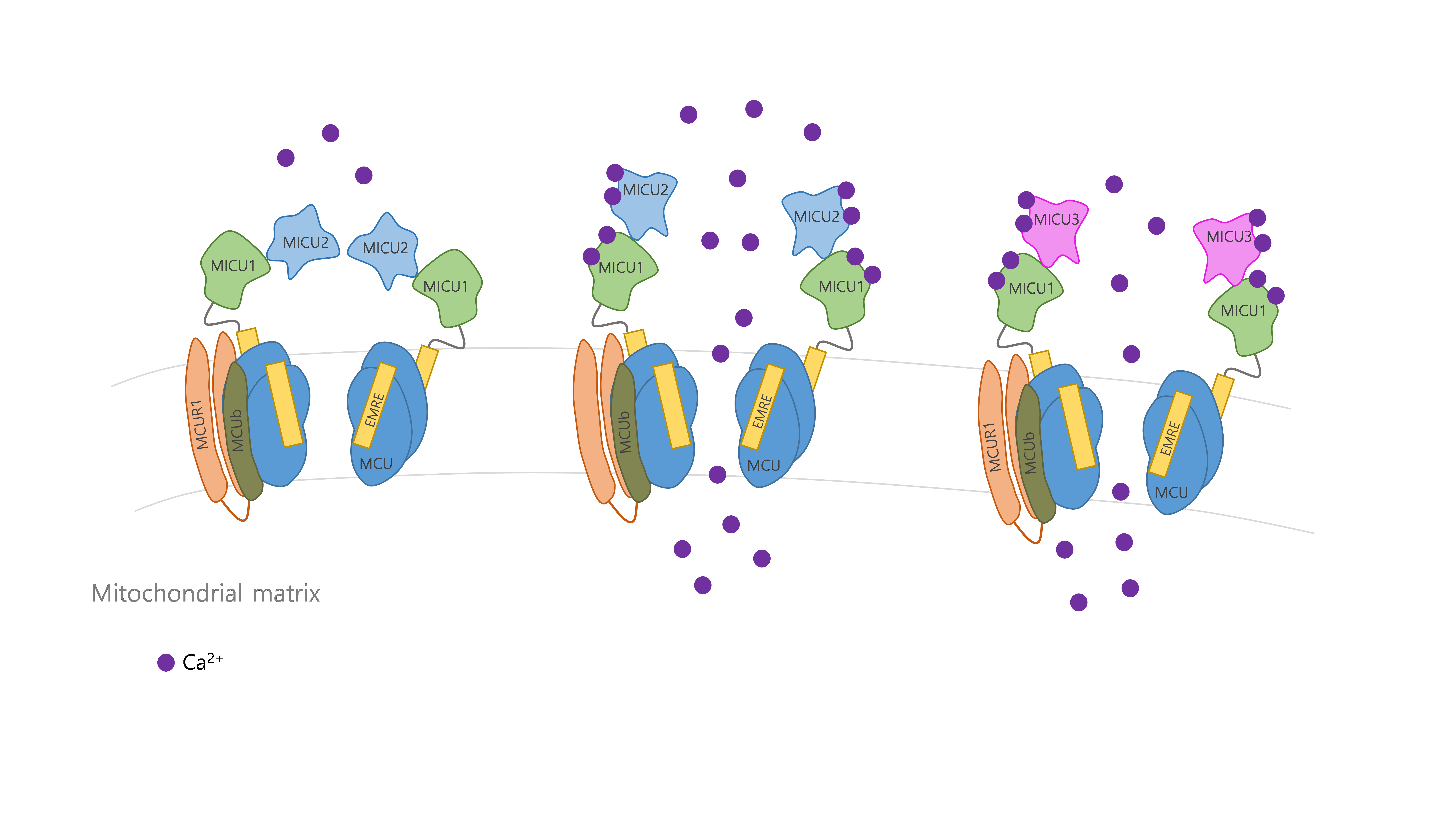

Supplement: Supplementary Figure 1 — MCU complex machinery. When the cytosolic Ca2+ level is low, MICU1/MICU2 heterodimer keeps MCU as a closed-form, whereas in high Ca2+ concentration, their conformational change helps MCU allow Ca2+ influx toward mitochondrial matrix. Otherwise, MICU1/MICU3 heterodimer has less gatekeeping function than MICU1/MICU2 dimer, which leads to opening the MCU complex in lower Ca2+ condition. [file Image_1.TIF]
